# Contribution of exome sequencing for genetic diagnostic in arrhythmogenic right ventricular cardiomyopathy/dysplasia

**DOI:** 10.1371/journal.pone.0181840

**Published:** 2017-08-02

**Authors:** Joel Fedida, Veronique Fressart, Philippe Charron, Elodie Surget, Tiphaine Hery, Pascale Richard, Erwan Donal, Boris Keren, Guillaume Duthoit, Françoise Hidden-Lucet, Eric Villard, Estelle Gandjbakhch

**Affiliations:** 1 INSERM, UMR_S1166, ICAN, Hôpital Pitié-Salpêtrière, Paris, France; 2 AP-HP, Groupe Hospitalier Pitié-Salpêtrière, Institut de Cardiologie, Paris, France; 3 AP-HP, Groupe Hospitalier Pitié-Salpêtrière, Service de Biochimie Métabolique, Unité de Cardiogénétique et Myogénétique, Paris, France; 4 AP-HP, Groupe Hospitalier Ambroise Paré, Département de Cardiologie, Boulogne, France; 5 AP-HP, Groupe Hospitalier Pitié-Salpêtrière, Centre de référence des maladies cardiaques héréditaires, Paris, France; 6 Département de Cardiologie, Hopital Pontchaillou, Rennes, France; King Abdulaziz University Hospital, SAUDI ARABIA

## Abstract

**Background:**

Arrhythmogenic Right Ventricular Cardiomyopathy/Dysplasia (ARVC/D) is an inherited cardiomyopathy mainly caused by heterozygous desmosomal gene mutations, the major gene being *PKP2*. The genetic cause remains unknown in ~50% of probands with routine desmosomal gene screening. The aim of this study was to assess the diagnostic accuracy of whole exome sequencing (WES) in ARVC/D with negative genetic testing.

**Methods:**

WES was performed in 22 patients, all without a mutation identified in desmosomal genes. Putative pathogenic variants were screened in 96 candidate genes associated with other cardiomyopathies/channelopathies. The sequencing coverage depth of *PKP2*, *DSP*, *DSG2*, *DSC2*, *JUP* and *TMEM43* exons was compared to the mean coverage distribution to detect large insertions/deletions. All suspected deletions were verified by real-time qPCR, Multiplex-Ligation-dependent-Probe-Amplification (MLPA) and cGH-Array. MLPA was performed in 50 additional gene-negative probands.

**Results:**

Coverage-depth analysis from the 22 WES data identified two large heterozygous *PKP2* deletions: one from exon 1 to 14 and one restricted to exon 4, confirmed by qPCR and MLPA. MLPA identified 2 additional *PKP2* deletions (exon 1–7 and exon 1–14) in 50 additional probands confirming a significant frequency of large *PKP2* deletions (5.7%) in gene-negative ARVC/D. Putative pathogenic heterozygous variants in *EYA4*, *RBM20*, *PSEN1*, and *COX15* were identified in 4 unrelated probands.

**Conclusion:**

A rather high frequency (5.7%) of large *PKP2* deletions, undetectable by Sanger sequencing, was detected as the cause of ARVC/D. Coverage-depth analysis through next-generation sequencing appears accurate to detect large deletions at the same time than conventional putative mutations in desmosomal and cardiomyopathy-associated genes.

## Introduction

Arrhythmogenic Right Ventricular Cardiomyopathy/Dysplasia (ARVC/D) is mainly caused by heterozygous mutations in genes encoding the main cardiac desmosome components (so-called desmosomal genes hereafter): *PKP2*, which is the major gene, *DSP*, *JUP*, *DSG2* and *DSC2* [1,2]. Other non desmosomal genes such as *TMEM43*, *RYR2*, *LMNA*, *PLN* or *DES* have been associated with atypical or typical forms of ARVC/D, suggesting genetic and clinical overlaps with dilated cardiomyopathy or catecholergic polymorphic ventricular tachycardia. International Task Force criteria, on which ARVC/D diagnosis is currently based, lack sensitivity in early phase of the disease [3,4]. However, the risk of sudden cardiac death is present throughout all stages, including the early phase. Predictive genetic testing is thus highly important to set-up cardiac follow-up and preventive strategies such as sport restriction in relatives at risk [5]. However, in families with negative genetic screening, identification of relatives at risk remains difficult, as ARVC/D diagnosis remains highly challenging due to a broad clinical spectrum of the disease and age- and gender-dependent penetrance.

Until recently, routine molecular screening of ARVC/D probands was usually performed using Sanger sequencing or High Resolution Melt (HRM) detection of desmosomal genes coding sequences. This approach allows the detection of the genetic cause in only ~50% of cases [6]. Several factors can explain negative genetic screening: (a) desmosomal mutations undetected by Sanger sequencing such as large *PKP2* deletions [7,8] (b) phenotype misclassification[9], (c) mutations in genes not known as causal in ARVC/D [10–14].

We hypothesized that next generation sequencing (NGS) represented in this study by whole exome sequencing (WES), could improve genetic diagnosis of ARVC/D by allowing the detection of new genetic causes or mechanisms for this rare cardiomyopathy. We focused our analysis on the detection of large deletions in desmosomal genes hardly detected by conventional Sanger sequencing, and on putative mutations in a large panel of genes associated with other inherited cardiomyopathies and arrhythmias.

## Material and methods

### Patient selection and clinical data

ARVC/D diagnosis was made according to Task Force criteria by a multidisciplinary consensus expert team [3]. We selected for WES: patients above 18 years with a diagnosis of familial ARVC/D (at least one affected first degree relative) or histologically proven ARVC/D and with no disease-causing mutations in known desmosomal genes (*PKP2*, *DSG2*, *DSP*, *DSC2*, *JUP)* with standard Sanger sequencing at the time of the study. One proband carried a variant of unknown significance in *DSG2* (p.Thr335Ala variant at homozygous state) and two others carried a rare benign polymorphism (*DSP* p.Gly2568Ser and *DSC2* p.Gly790del). All patients gave their informed consent for genetic study and research purposes; the study was performed according to the declaration of Helsinski and was approved by local ethics committee, the Pitié-Salpêtrière Hospital ethical committee. Overall, WES was performed in 23 subjects: 16 index cases with familial history of ARVC/D, one family with 3 affected family members and a healthy relative used as internal control and 3 sporadic cases with severe ARVC/D. We secondarily selected 50 additional ARVC/D unrelated probands with previous negative genetic testing for Multiplex-Ligation-dependent-Probe-Amplification (MLPA) analysis. We collected the following clinical data for each patients and available relatives: symptoms, 12-leads ECG, signal averaged-ECG, 24h-Holter monitoring, exercise test, cardiac imaging (echocardiography, cardiac MRI and contrast angiography), histology when available and familial history.

### Exome sequencing and coverage depth analysis (details in S1 Methods)

WES was performed by targeted capture using the “Agilent SureSelect All Exon v5 50Mb kit” and 75 bases paired-end sequencing with Illumina HiSeq 2000 (Integragen). Copy number variation (CNV) was analyzed using the DNACopy (Bioconductor) package furnished by Integragen. The coverage depth of each exon of *PKP2*, *DSG2*, *DSP*, *JUP* and *DSC2* was analyzed on 25 base pairs windows with the IGV software (https://www.broadinstitute.org/igv*)*. The depth of coverage distribution for each exon, reflecting copy number of genomic targets, was normalized to the mean coverage in the whole cohort (23 patients). Heterozygous deletion was suspected if normalized coverage was less than one standard deviation (SD) of the mean coverage distribution for the whole cohort.

### Cardiomyopathy panel description

All the genes known to be associated with inherited cardiomyopathies were selected (detail in S2 Method). We selected rare variants with depth coverage > 10 and a minor allelic frequency (MAF) < 0.05% in the control databases 1000 genomes (http://1000genomes.org) and EXAC (http://exac.broadinstitute.org). Rare variants were considered as putative mutations in case of (a) radical mutations (nonsense, ins/del, splice-site); (b) missense mutations predicted as disease-causing by at least 4 over 5 prediction softwares (Polyphen-2, SIFT, Mutation Taster,mutation assessor and CADD score > 20). Criteria for classifying variants as pathogenic or benign were assessed for each variant according to the ACMG standards and guidelines [15]. Variants classified as pathogenic by 4 over 5 prediction softwares were classified PP3, otherwise they were classified BP4. Variants absent in control databases EXAC and 1000 genomes were classified PM2. Variants with a MAF >0.015% in EXAC (which corresponds to the higher frequency of *PKP2* disease-causing mutations in EXAC) were classified BS1. *In silico* analysis were performed using the wANNOVAR tool (http://wannovar.usc.edu).

### Quantitative real-time PCR (qPCR)

Quantitative real-time PCR (qPCR) assays using primers specific for the *PKP2* exons 1, 3, 4, 5 and 13 were performed to validate large deletions (details in S1 Table).

### MLPA, microarray and targeted capture sequencing

MLPA was performed using the SALSA MLPA168 ARVC-*PKP2* commercial kit from MRC-Holland^®^ on an ABI-Prism 3130. The microarray was performed with a HumanOmniExpress-24v1.0 chip (Illumina) and analysed with the Illumina GenomeStudio V2011.1 software. Targeted Capture sequencing of *PKP2* was performed with the Trusight Cardiomyopathy sequencing panel (Illumina) on a Miseq (Illumina).

### Immunofluorescence study

Heart tissues samples (right and left ventricles, directly frozen in isopentane) were collected from the proband U.1 explanted heart, as described previously [16]. Patient gave his informed consent for the use of tissue samples for research purpose. Five μm sections were fixed in paraformaldehyde/ cold methanol (-20°C) and co-imuno-labelled with primary antibodies anti-*PKP2* (PKP2-518, Progen, 1/4). Monolayers were directly visualized by fluorescent microscopy with an Olympus IX50 microscope and 3D deconvolution was performed using the Metamorph software (Roper Scientific).

## Results

### WES coverage depth analysis

**Fig 1:**
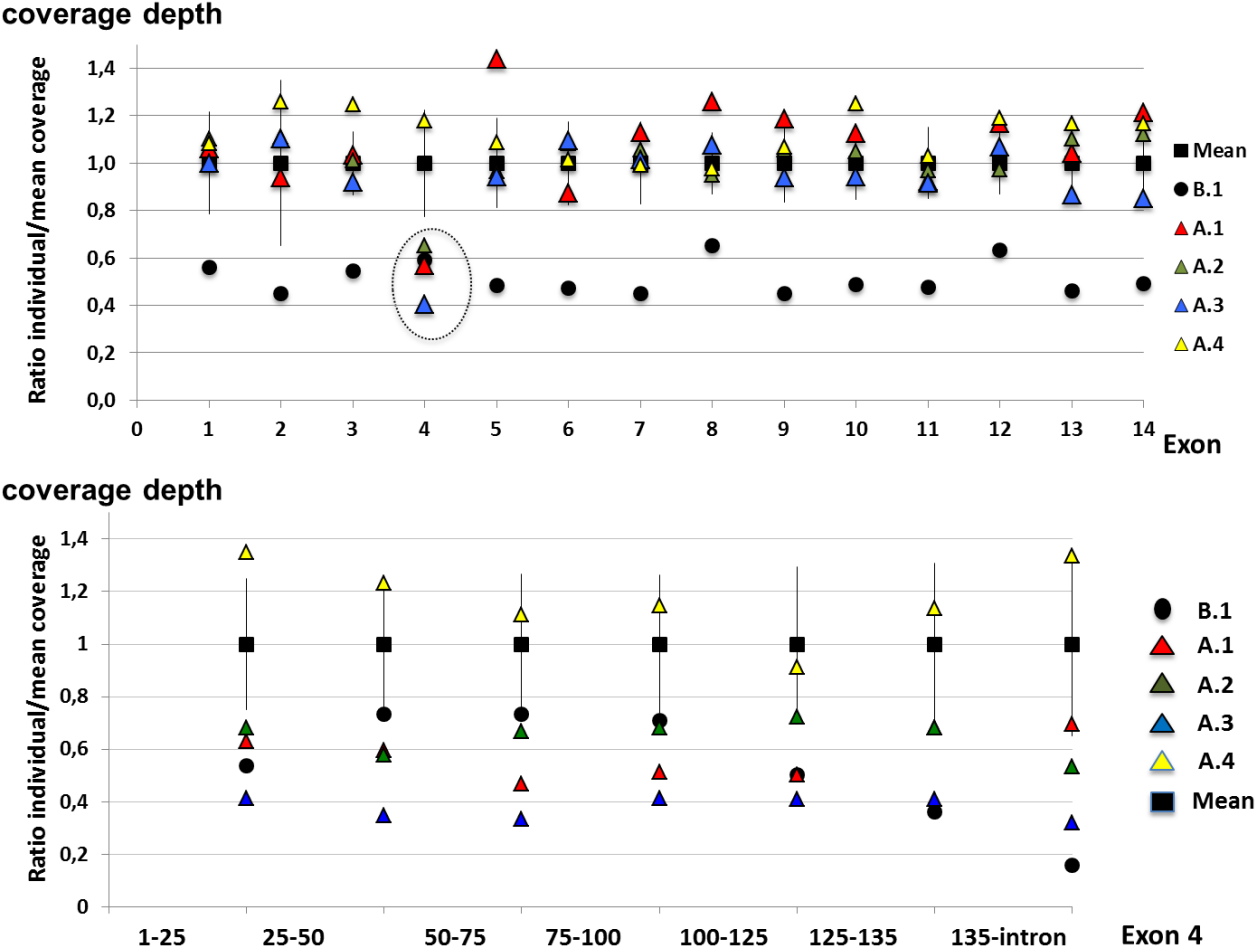
***PKP2* exons coverage depth analysis from WES in individuals from family A and B.1.** The coverage depth of all *PK2* exons was under standard deviation of the mean coverage in individual B1 indicating a probable deletion of the whole *PKP2* gene. Coverage depth analysis showed a coverage reduction restricted to exon 4 in affected individuals from family A (dashed circle).

Summary of WES run including average coverage per sample and remarkable values (number of missense variant, non-sense variant and Indel present with a frequency less than 0,1% of the control cohort) are represented in S2 Table. For all patients, the exome coverage depth percentage of at least 10 reads were above 95%.The coverage depth of *PKP2* exons was > 35 reads for all exons except exon 1 and 2 that displayed a low coverage depth (9.7±2.1 and 3.0±1.1 reads respectively, S3 Table). Normalized coverage distribution analysis showed low coverage under one SD for all *PKP2* exons in individual B.1 (45 to 65% of the mean coverage value) suggesting whole *PKP2* heterozygous deletion. In family A, we observed a low coverage of exon 4 in the three affected subjects (A.1, A.2 and A.3, from 41 to 65% of the mean coverage value) suggestive of deletion restricted to exon 4 (Fig 1). Exon 4 coverage depth of the healthy relative (individual A.4) was within SD values (118%). Coverage depth analysis of *JUP*, *DSC2*, *DSP*, *DSG2* and *TMEM43* did not identify CNV in these genes.

### MLPA, qPCR and cGH-Array analysis

Our rate of detection of large deletion in *PKP2* using NGS coverage was close to 10%. In order to confirm this finding and to better assess this frequency we performed MLPA in the 20 initial probands and in 50 additional unrelated ARVC/D probands with negative genetic screening in desmosomal genes. MLPA confirmed the heterozygous deletion restricted to exon 4 in family A and the heterozygous whole *PKP2* deletion in patient B.1. Furthermore, we identified by MLPA a complete *PKP2* deletion in two additional probands (U.1 and V.1). Overall, the frequency of large *PKP2* deletions was 5.7% (4/70) in ARVC/D with initial negative screening. No large deletions were detected in *JUP*, *DSG2*, *DSC2*, *DSP* or TMEM43 by MLPA, as observed by WES.

**Fig 2:**
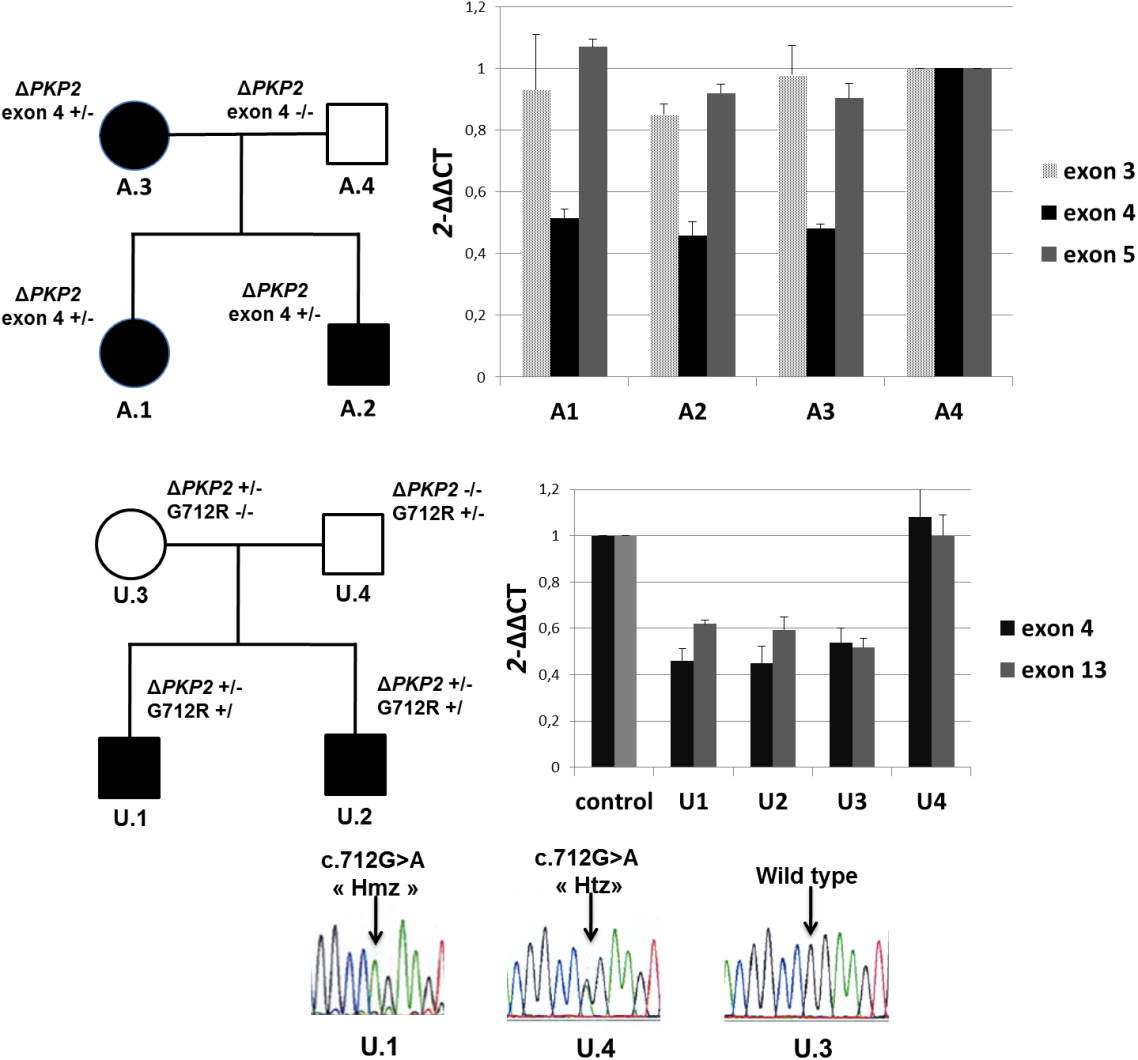
Pedigree and qPCR results from family A and U. qPCR showed a ~50% reduction of exon 4 *PKP2* DNA in affected individuals A.1, A.2 and A3 compared to the healthy father A.4 (used as a control). *PKP2* DNA quantification was normal in exon 3 and 5 for all individuals. In family U, qPCR showed a 50% reduction of *PKP2* DNA in exon 4 and 13 in affected individuals U.1 and U.2 and their mother U.3 whereas it was comparable to controls in father U.4. Individuals U.1 and U.2 also carried the p.Gly712Arg (G712R) rare *PKP2* variant that falsely appeared in the Sanger chromatogram as homozygous (HmZ) instead of hemizygous because of the absence of the other *PKP2* allele. This variant was inherited from the father, in absence of familial history of consanguinity, in whom it appeared heterozygous(Htz).

All *PKP2* deletions identified through coverage depth analysis and MLPA were confirmed using qPCR. The relative copy number of *PKP2* exon 4 was about 0.5 in all affected individuals from family A compared with the healthy control but the relative copy numbers of exon 3 and 5 were about 1. This confirmed the presence of the heterozygous deletion of exon 4 that cosegregated with the phenotype in the family (Fig 2 and S4 Table). In families B and U, real-time qPCR indicated a relative copy number of about 0.5 for *PKP2* exon 3, 4 and 13 in affected probands and relatives compared with the control sample, confirming the large heterozygous *PKP2* deletion (Fig 2 and S5 Table). In both families, the large deletion was inherited from the asymptomatic mother who has no ARVC criteria of diagnosis on cardiac evaluation, and present in the affected brother (Table 1).

**Table 1:** Clinical data of probands and relatives carrying a large *PKP2* heterozygous deletion.

Family	Patient	Familial status	Sex	Age at diagnosis	Family history	TWI	Epsilon waves	SA-ECG	Arrhythmia	RV	LV	TF criteria	Clinical status	Deletion	Deletion bonds
**A**	**1**	Proband	F	29	SCD gd-father at 30 (min)	V1-V3 (Maj)	abs	abs	RVOT VT (min)	Maj	abs	2Maj/2min	Affected	Exon 4	Arr[hg19] 12p11.21 [32,979,318–33,062,23]x1
	**2**	Brother	M	21	Maj	V1-V3 (Maj)	abs	abs	abs	Maj	abs	3Maj	Affected	Exon 4	
	**3**	Mother	F	52	Maj	V1-V3 (Maj)	abs	abs	>500 PVC (min)	min	abs	2Maj/2min	Affected	Exon 4	
**B**	**1**	Brother	M	37	Maj	abs	Maj	+	RVOT VT (min)	Maj	abs	3Maj/1min	Affected	Exon 2–14	Arr[hg19] 12p11.21 [32,943,679–33,049,780]x1
	**2**	Proband	M	45	abs	V1-V3 (Maj)	Maj	+	>500 PVC (min)	Maj	abs	3Maj/1min	Affected	Exon 2–14	
	**3**	Mother	F	73	Maj	V1-V3 (Maj)	abs	abs	NSVT (min)	Maj	abs	3Maj/1min	Affected	Exon 2–14	
**U**	**1**	Proband	M	17	abs	V1-V6 (Maj)	Maj	+	SCD, Sustained VT (min)	Maj	+	3Maj/1min	Affected	Exon 1–14 + *PKP2* G712R	Arr[hg19] 12p11.21 [32,935,752–33,064,825]x1
	**2**	Brother	M	23	Maj	abs	Maj	abs	abs	abs	abs	2Maj	Affected	Exon 1–14 + *PKP2* G712R	
	**3**	Mother	F		Maj	NA	NA	NA	NA	NA	NA	NA	NA	Exon 1–14	
**V**	**1**	Proband	M	22	abs	V1-V3 (Maj)	abs	abs	Sustained VT (min)	min	abs	1Maj/2min	Affected	Exon 1–7	Arr[hg19] 12p11.21 [32,979,318–33,062,23]x1

M: male; F: female; Maj/min/abs: major/minor/absent criterion of the International 2010 ARVC Task Force; TF criteria: task force criteria; TWI: T wave inversion; SAECG: signal-average ECG; RVOT VT: ventricular tachycardia from right ventricular outflow tract; PVC: premature ventricular complex; RV: right ventricle; LV dys: left ventricle dysfunction; SCD: sudden-cardiac-death; NSVT: non-sustained VT; NA: non-available data. Deletions bonds are given according to the ISCN 2013 guidelines (ref GRCh37/hg19).

**Fig 3:**
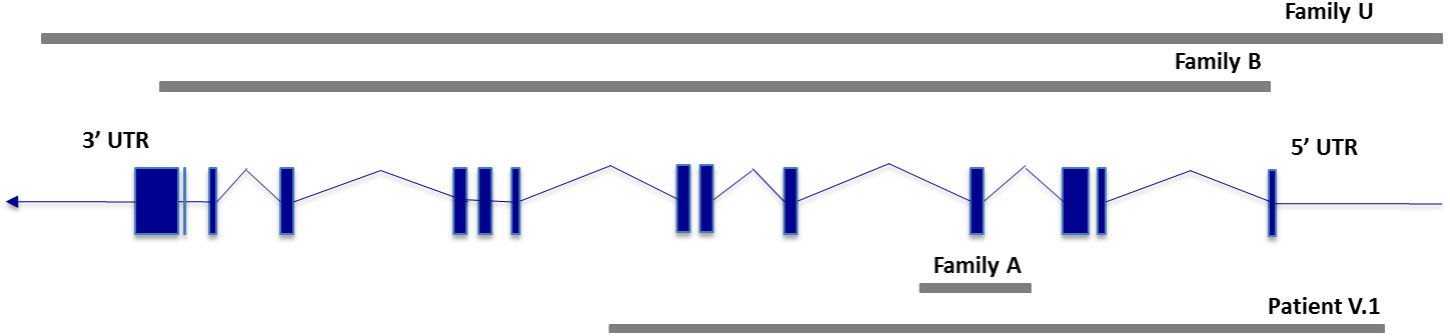
Schematic view of *PKP2* deletions.

The microarray analysis confirmed the three complete *PKP2* deletions in individuals B.1, U.1 and V.1 (details in Table 1 and Fig 3) but lacked sensitivity to detect the deletion restricted to exon 4 in proband A.1 due to a unique informative SNP of this exon (which had a negative log ratio).

Targeted capture and subsequent NGS of *PKP2* exons was performed in patient U.1 confirming a 50% coverage reduction for all exons (S1 Fig).

### Clinical data of *PKP2* large deletion carriers

Phenotype of individuals carrying a large *PKP2* deletion is provided in Table 1. All of them displayed a definite ARVC/D diagnosis. Patient U.1 developed severe ARVC/D with bi-ventricular involvement and heart failure leading to heart transplantation. This patient was a compound heterozygous for the large *PKP2* deletion (inherited from his mother) and the rare *PKP2* p.Gly712Arg variant of unknown pathogenicity (allele frequency 0.0024%) inherited from his father in absence of familial history of consanguinity, which therefore appeared as “homozygous” instead of hemizygous in Sanger sequencing (Fig 2). The *Mutation Taster* and *Polyphen-2* prediction software classified this rare variant as deleterious, however with probable incomplete penetrance in a heterozygous context. The immunofluorescence staining of *PKP2* in cardiac tissue from the proband U.1 explanted heart showed normal *PKP2* location at intercalated disks (S2 Fig).

### Cardiomyopathy associated genes analysis

96 genes previously associated with various cardiomyopathies/channelopathies were screened for putative mutation. 41 rare variants with MAF< 0.05% were present in 20 different genes (*ANK2*, *CACNB2*, *COX15*, *DSP*, *EYA4*, *FLNC*, *KCNE1*, *KCNH2*, *KCNJ5*, *KCNQ1*, *MYBPC3*, *MYH6*, *MYLK2*, *MYOM1*, *NEXN*, *PSEN1*, *RBM20*, *SCN1B*, *SOS1*, *TTN*) (see S6 Table—Rare cardiomyopathy variants with AGMG score). However, only 4 heterozygous rare genetic variants met at least one or two criteria for pathogenicity without benign criteria:: p.Arg356Gly in *EYA4*; p.Arg761Gln in *RBM20*, p.Tyr189Cys in *PSEN1*, p.Gly174Ser in *COX15* which were identified in four unrelated probands. The clinical data and the detailed list of putative mutations are available in Tables 2 and 3. None of these patients displayed an extra-cardiac involvement. The *EYA4* and *RBM20* variants were also present in the affected father of the proband. The *COX15* variant did not segregate with the disease within the family and was therefore unlikely to be disease causing. No segregation data were available for the *PSEN1* variant. The *EYA4*, *PSEN1* and *RBM20* variants are classified as variants of uncertain significance according to the ACMG guidelines in absence of large familial segregation study or functional study.

**Table 2:** Clinical data of probands carrying putative pathogenic mutations.

Patient	Familial status	Sex	Age at diagnosis	Family history	TWI	Epsilon waves	SA-ECG	Arrhythmia	RV	LV dys	TF criteria	Clinical status	Suspected gene
QT4286	Proband	F	17	abs	V1-V5 (Maj)	abs	+ (min)	> 500 PVC (min)	Maj	abs	2Maj/ 1min	Affected	*EYA4*
QT7052	Proband	F	21	abs0	V1-V4 (Maj)	abs	abs	NSVT (min)	Maj	abs	2Maj/ 1min	Affected	*RBM20*
1–46767	Proband	M	34	+ (min)	0	abs	+ (min)	NSVT (min)	Borderline	abs	3min	Borderline	*COX15*
QT4029	Proband	M	46	abs	V1-V4 (Maj)	abs	+ (min)	Sustained VT (Maj)	Maj	abs	3Maj/ 1min	Affected	*PSEN1*

M: male; F: female; Maj/min/abs: major/minor/absent criterion of the International 2010 ARVC Task Force; TF criteria: task force criteria; TWI: T wave inversion; SAECG: signal-average ECG; RVOT VT: ventricular tachycardia from right ventricular outflow tract; PVC: premature ventricular complex; RV: right ventricle; LV dys: left ventricle dysfunction; SCD: sudden-cardiac-death; NSVT: non-sustained VT; NA: non-available data.

**Table 3:** Detailed list and characteristics of putative mutations.

Patient	Origin	*Gene*	Protein (c.DNA)	Allele	1000 Genome MAF	ExAC MAF	SIFT	Polyp- 2	Mutation Taster	Mutation Assessor	CADD score	Segregation	ACMG criteria	Literature-reported associated phenotypes
QT4286	Caribbean	*EYA4 (NM_172105)*	p.Arg356Gln (c.1085G>A) rs762144530	HtZ	absent	0.002% (absent*)	D	D	D	M	36	Yes (affected father)	PP3, PP1	Dominant DCM and hearing loss[17]
QT7052	Caucasian	*RBM20 (NM_001134363)*	p.Arg761Qln (c.2282G>A) rs556897484	HtZ	0.020%	0.005%	D	D	D	L	22.5	Yes (affected father)	PP3, PP1	Dominant DCM[18–19]
1–46767	Caucasian	*COX15 (NM_004376)*	p.Gly174Ser (c.520G>A) rs763842058	HtZ	absent	0.005%	D	D	D	H	36	no	PP3, BS4	Recessive Leigh Syndrome/cardio-encephalopathy
QT4029	Caribbean	*PSEN1 (NM_000021)*	p.Tyr189Cys (c.566A>G) rs556147068	HtZ	0.020%	0.002% (0.01%*)	D	D	D	M	21.3	NA	PP3	Dominant DCM [20]

SIFT: D: damaging, T: tolerant; Polyphen-2: D: damaging, P: polymorphism; Mutation taster: D: damaging; Mutation Assessor: probability of pathogenic mutation: L: low, M: medium, H: High. DCM: dilated cardiomyopathy; RCM: restrictive cardiomyopathy, HCM: hypertrophic cardiomyopathy. HmZ: homozygous; HtZ: heterozygous. MAF: minor allele frequency. NA: not available.

*: EXAC MAF in African population.

CNV in cardiomyopathy genes were also screened (results in S7 Table). Putative large deletion of *NEXN* and *SDHA* and duplication of *ACTA1* were identified in family A where a *PKP2* large deletion was also found. These CNVs did not segregate with the disease within the family and were therefore considered as polymorphisms. A duplication of *TNNT2* was also identified in one proband but was considered as a probable polymorphism because of the high frequency of *TNNT2* duplication in control databases (S7 Table). A putative large deletion of *ABCC9* was identified in one proband and was considered of unknown significance because of its low frequency in controls.

## Discussion

This study reports for the first time the identification of large *PKP2* deletions through coverage depth analysis and the added value of WES for genetic diagnosis in ARVC/D.

### Accuracy of WES for detection of large *PKP2* deletions

WES data can be used to check large CNVs in ARVC/D patients with no disease-causing mutations in known desmosomal genes. WES allowed the diagnosis of large *PKP2* deletions in two independent probands. These deletions were undetected by standard Sanger sequencing. Overall, we observed a 5.7% frequency of large deletions in ARVC/D with previous negative genotyping in desmosomal genes. This frequency is higher than previously reported, as frequency of large deletions ranged between undetected in a Danish cohort and 2 to 3% in a Dutch cohorts [21,22]. However, the higher *PKP2* deletion frequency observed in our study is probably linked to the selective inclusion of cases with no detected mutation after a standard primary mutation screening. Phenotypes associated with large *PKP2* deletions in our cohort were typical ARVC/D phenotypes and all fulfilled International task force diagnosis for ARVC/D. The sole patient with a severe phenotype also carried a hemizygous rare missense *PKP2* variant (p.Gly712Arg) possibly worsening the expression of the disease. This hypothesis is supported by previous studies showing the modifying role of rare *PKP2* missense variants [23,24]. Such variants would have little effect in the presence of a normal allele but express its pathogenicity in a hemizygous context. Due to the associated heterozygous large *PKP2* deletion, the rare missense variant appeared as “homozygous” by Sanger sequencing. Therefore, the identification of such rare homozygous variants in routine sequencing should raise suspicion for an underlying large deletion of the other allele.

Our results suggest that large *PKP2* mutations are a significant cause of ARVC/D undetected by routine Sanger sequencing worthy to be screened in routine molecular diagnosis. NGS presents a better cost/effectiveness ratio and progressively substitutes to Sanger sequencing for routine molecular screening of inherited cardiomyopathies. Our study shows that the coverage depth analysis of NGS appears effective to detect large *PKP2* mutations, including those restricted to a single exon as in family A. A careful analysis is however necessary to detect single exon deletions. All suspected deletions were confirmed by qPCR and MLPA, demonstrating the sensitivity of this method. However, coverages of exon 1 and 2 in WES were particularly low (< 10 reads) due to GC-rich contents. Lack of sensitivity of WES to detect early exons deletions in *PKP2* due to low coverage of these exons might be circumvent using targeted capture based resequencing as strongly suggested by the high coverage (169 to 325 reads) observed when it was applied to U.1 patient’s DNA (S1 Fig). Although no insertion CNV was identified by coverage depth analysis in desmosomal genes, we cannot exclude that the mean coverage values obtained by WES preclude accurate detection of these variations. The microarray method is highly accurate to detect large deletion >300 kb but lacked sensitivity to detect the deletion restricted to a single exon, as observed in family A. MLPA was accurate to detect large deletions but the probes targeting the exon 1, 2 and 12 are located outside the exons and therefore could miss a deletion restricted to one exon located outside the probes. Advantages and weaknesses of the different techniques are presented in S8 Table. Overall, targeted capture sequencing of *PKP2* appears as the most appropriate technique as it has probably the best sensitivity for detection of large deletions through coverage depth analysis and is able to detect point or small ins/del mutations at the same time [25].

### Genetic overlap with other cardiomyopathies

The analysis of 96 genes associated with various inherited cardiomyopathies identified 4 putative pathogenic variants in *EYA4*, *COX15*, *PSNE1* and *RBM20*. The *COX15* variant did not segregated with the disease and was unlikely to be disease-causing. One patient with severe biventricular involvement that progressed to end-stage heart failure and heart transplantation carried a deleterious *PSEN1* missense variant (p.Tyr189Cys). Mutations in this gene, associated with Alzheimer disease, were previously reported in severe progressive dilated cardiomyopathy (DCM) [20]. Another proband with classical ARVC/D phenotype associating RV involvement, T-wave inversion in precordial leads, epsilon wave and RV tachycardia carried a rare deleterious *RBM20* missense variant. *RBM20* mutations located within a highly-conserved arginine/serine (RS)-rich region gene were previously identified in familial DCM [18,19]. However, the rare variant p.Arg761Qln is located outside this mutational hot-spot and thus remains of unknown significance. Furthermore, the *PSEN1* and the *RBM20* variant frequencies were 0.02% in the 1000 genome database, which appears high in the setting of this rare disease. Another missense variant predicted as deleterious was found in *EYA4*, a gene associated with progressive DCM and hearing loss [17]. However, no hearing anomaly was documented in the family. Although these putative variants were retained through highly stringent process, it remains difficult to conclude on their pathogenicity in absence of functional and large familial segregation studies, whereas the *RBM20* and the *EYA4* variants were also identified in the affected relative. A putative deletion of *ABCC9* was also identified in one ARVC/D proband and was considered of unknown significance. *ABBC9* mutations were previously identified in DCM patients with ventricular arrhythmias [26]. Overlapping phenotypes between DCM and right ventricular cardiomyopathies were described with other genes, such as *LMNA* and *PLN*. We cannot exclude that some of these “new” candidate genes, previously associated with DCM, could be associated with ARVC/D phenotype. Further analyses are therefore needed to determine the role of these genes in ARVC/D: screening in larger cohorts, large familial segregation and functional studies but also additional WES studies in other ARVC/D cohorts.

## Conclusion

In conclusion, we observed a rather high 5.7% frequency of large deletions in ARVC/D as the cause of gene-negative ARVC/D undetected by conventional Sanger sequencing, suggesting need for systematic routine screening of such mutations. Coverage depth analysis through NGS (targeted capture sequencing or WES), which has become the reference sequencing method, appears as an accurate method to detect such deletions. This finding reinforces haploinsufficiency as the major pathophysiological mechanism of *PKP2* mutations in ARVC/D. We also identified putative deleterious genetic variants in four associated-cardiomyopathy genes, possibly expanding the genetic spectrum of ARVC/D.

## Supporting information

S1 Fig Coverage depth analysis of *PKP2* obtained by targeted capture in patient U.1. 50% coverage reduction was observed in patient U.1 (red line) compared to controls, indicating a probable *PKP2* heterozygous deletion of all exons (mean *PKP2* coverage ranged between 169 and 325 reads in controls).
(TIF)

S2 Fig Immunofluorescence staining of *PKP2* in the right ventricle from explanted heart from patient U.1 carrying the whole *PKP2* deletion and the G712R *PKP2* variant, showing correct localization of *PKP2* at intercalated disks. (TIF)

S1 Table Primers used for *PKP2* and *TBP* qPCR. (DOCX)

S2 Table Average coverage of the 23 patients. Number of missense variant, non-sense variant and Indel present with a frequency less than 0,1% of the control cohort are represented here.
(DOCX)

S3 Table Details of coverage depth for each *PKP2* exon. (DOCX)

S4 Table qPCR values of family A. Mean ±SD of triplicates. Patient A4 serves as control.
(DOCX)

S5 Table qPCR values of family U. Mean ±SD of triplicates. Patient A4 serves as control.
(DOCX)

S6 Table Rare cardiomyopathy variants with AGMG score. (XLSX)

S7 Table List of CNVs in cardiomyopathy genes detected by the DNACopy (Bioconductor) package and frequency of exonic CNV in controls (from data of the database of genomic variants at http://dgv.tcag.ca). (DOCX)

S8 Table Comparison of technique for detection of large PKP2 deletion. (DOCX)

S1 Method Whole exome sequencing and bioinformatic analysis. (DOCX)

S2 Method List of genes known to be associated with inherited cardiomyopathies. (DOCX)

## Abbreviations

ARVC/D: Arrhythmogenic Right Ventricular Cardiomyopathy/Dysplasia
CNV: Copy Number Variation
DCM: dilated cardiomyopathy
HRM: High Resolution Melt
MLPA: Multiplex-Ligation-dependent-Probe-Amplification
NGS: Next generation sequencing
qPCR: Quantitative real-time PCR
RV: Right ventricle
WES: Whole exome sequencing
