# Supplementary figures and images for: Contribution of exome sequencing for genetic diagnostic in arrhythmogenic right ventricular cardiomyopathy/dysplasia
Source: PLoS One. 2017 Aug 2;12(8):e0181840. doi: 10.1371/journal.pone.0181840 (PMC5540585; doi:10.1371/journal.pone.0181840)

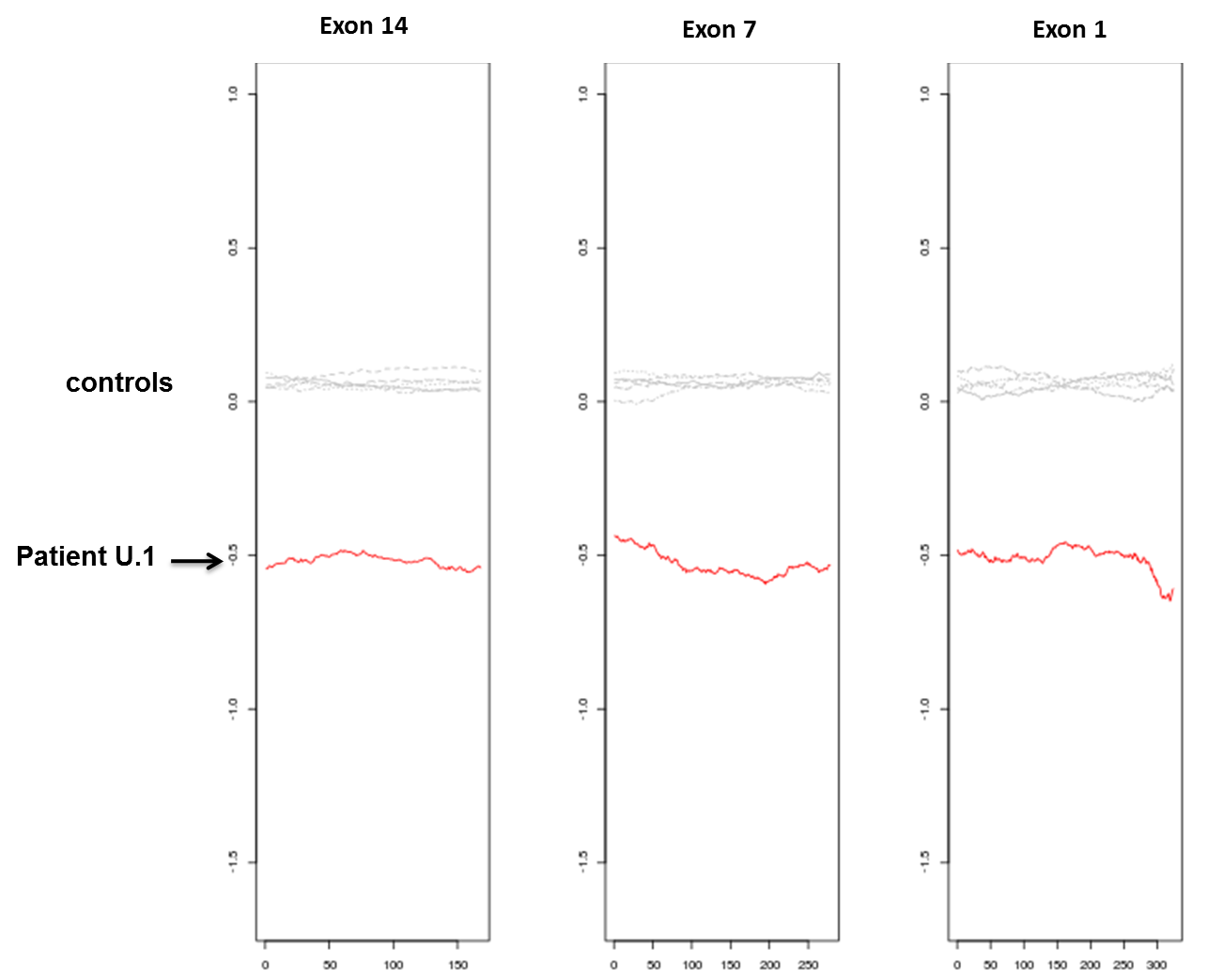

Supplement: S1 Fig — 50% coverage reduction was observed in patient U.1 (red line) compared to controls, indicating a probable PKP2 heterozygous deletion of all exons (mean PKP2 coverage ranged between 169 and 325 reads in controls). (TIF) [file pone.0181840.s001.tif]

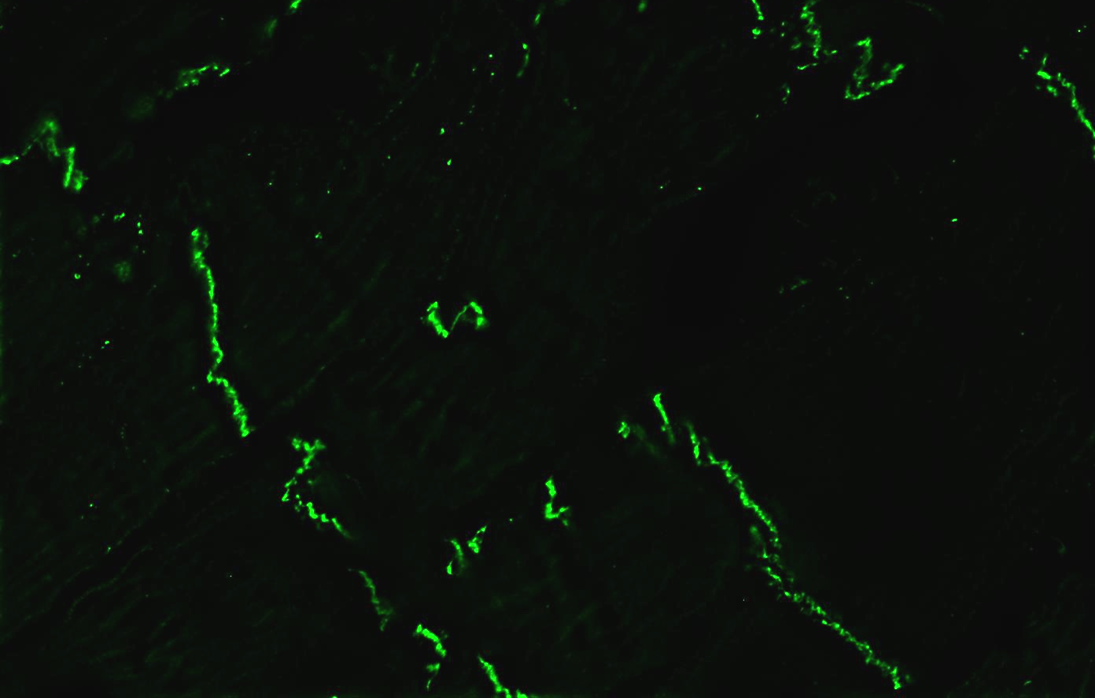

Supplement: S2 Fig — (TIF) [file pone.0181840.s002.tif]
