# Supplementary material for: Contribution of exome sequencing for genetic diagnostic in arrhythmogenic right ventricular cardiomyopathy/dysplasia
Source: PLoS One. 2017 Aug 2;12(8):e0181840. doi: 10.1371/journal.pone.0181840 (PMC5540585; doi:10.1371/journal.pone.0181840)
Supplement: S1 Table — (DOCX) [file pone.0181840.s003.docx]

**S1 Table. Primers used for *PKP2* and *TBP* qPCR.**

| Primers | Sequence 5’ 🡪 3’ | Tm |
| --- | --- | --- |
| PKP2-Exon1 Forward | CGGCCAGACAGTCAAGAG | 62°C |
| PKP2-Exon1 Reverse | CTCCACTCACCGTTGCC | 62°C |
| PKP2-Exon2 Forward | GTTCCTGAGTATGTCTACAACC | 60°C |
| PKP2-Exon2 Reverse | GATATCCTACCTTTAGCATGTCATA | 60°C |
| PKP2-Exon3 Forward | GATTACCAGTACAGCCAGAGAAG | 62°C |
| PKP2-Exon3 Reverse | CGATCTCGGAACGAGCATATC | 62°C |
| PKP2-Exon4 Forward | GCTGAATCTGTGCCTGTTTG | 62°C |
| PKP2-Exon4 Reverse | GAAAGTAGCTGCAGCAGAAATC | 62°C |
| PKP2-Exon5 Forward | GTGGCATCCTCAAGCTTCT | 62°C |
| PKP2-Exon5 Reverse | CCACCTCCAATTTGTTGTCATT | 62°C |
| PKP2-Exon13 Forward | AGCAGTTGAGGAGCGAAGAG | 62°C |
| PKP2-Exon13 Reverse | TTGTTGGAGGCATAGCTGAA | 62°C |
| TBP-Exon2 Forward | CTGTTTCTTGGCGTGTGAAG | 62°C |
| TBP-Exon2 Reverse | CGCTGGAACTCGTCTCACTA | 62°C |

Amplification conditions consisted of initial denaturation step at 95°C for 3min, followed by 45 cycles at 95°C for 5s and at 62°C for 10min. Then elongation conditions consisted of 1 cycle at 95°C for 5s, 62°C for 1 min and 97°C continuous. TATA-binding protein (*TBP*) gene was used as internal reference to normalize the target gene copy number. Genomic DNA was amplified by real-time PCR on a Roche LightCycler^®^ 480 using the SYBR^®^ Green PCR Master Mix (DyNAmo™ HS SYBR^®^ Green qPCR Kit, Finnzymes, Espoo, Finland). Validation experiments were performed using the standard curve method with five serial dilutions of genomic DNA from control subjects. PCR efficiencies (E) for all exons wercalculated. Amplification efficiencies were identical and the relative gene copy number was calculated by the 2^-ΔΔCt^ method (*Livak KJ, Schmittgen TD. Analysis of Relative Gene Expression Data Using Real-Time Quantitative PCR and the 2−ΔΔCT Method. Methods. 2001 december; 25(4):402–8.)*
