# Supplementary material for: Contribution of exome sequencing for genetic diagnostic in arrhythmogenic right ventricular cardiomyopathy/dysplasia
Source: PLoS One. 2017 Aug 2;12(8):e0181840. doi: 10.1371/journal.pone.0181840 (PMC5540585; doi:10.1371/journal.pone.0181840)
Supplement: S2 Table — Number of missense variant, non-sense variant and Indel present with a frequency less than 0,1% of the control cohort are represented here. (DOCX) [file pone.0181840.s004.docx]

| Sample | Mean coverage/Target base (%) | | Exome coverage depth percentage (%) of at least 10 reads | Number of missense  with frequency  < 0,1% | Number of non-sense variant  with frequency  < 0,1% | Number of Indel with frequency  < 0,1% |
| --- | --- | --- | --- | --- | --- | --- |
| Patient B.1 | | 82 | 95 | 468 | 13 | 38 |
| Patient C | | 74 | 95 | 464 | 11 | 38 |
| Patient D | | 70 | 95 | 471 | 13 | 37 |
| Patient E | | 82 | 95 | 481 | 12 | 38 |
| Patient F | | 74 | 95 | 196 | 7 | 13 |
| Patient G | | 75 | 95 | 505 | 10 | 24 |
| Patient H | | 79 | 95 | 349 | 9 | 28 |
| Patient I | | 68 | 95 | 222 | 3 | 20 |
| Patient J | | 78 | 95 | 184 | 6 | 8 |
| Patient K | | 72 | 95 | 349 | 5 | 37 |
| Patient L | | 72 | 95 | 182 | 5 | 19 |
| Patient M | | 79 | 95 | 190 | 4 | 9 |
| Patient N | | 76 | 95 | 286 | 8 | 19 |
| Patient O | | 67 | 95 | 190 | 7 | 17 |
| Patient P | | 75 | 95 | 373 | 3 | 34 |
| Patient Q | | 68 | 95 | 182 | 5 | 18 |
| Patient R | | 69 | 95 | 480 | 10 | 52 |
| Patient S | | 80 | 95 | 224 | 5 | 19 |
| Patient T | | 77 | 95 | 211 | 5 | 18 |
| Patient A.1 | | 73 | 95 | 202 | 3 | 12 |
| Patient A.2 | | 82 | 95 | 354 | 8 | 36 |
| Patient A.3 | | 90 | 96 | 214 | 4 | 15 |
| Patient A.4 | | 77 | 95 | 406 | 10 | 42 |

S2 Table. Average coverage of the 23 patients. Number of missense variant, non-sense variant and Indel present with a frequency less than 0,1% of the control cohort are represented here.
