# Supplementary material for: Contribution of exome sequencing for genetic diagnostic in arrhythmogenic right ventricular cardiomyopathy/dysplasia
Source: PLoS One. 2017 Aug 2;12(8):e0181840. doi: 10.1371/journal.pone.0181840 (PMC5540585; doi:10.1371/journal.pone.0181840)
Supplement: S3 Table — (DOCX) [file pone.0181840.s005.docx]

**S3 Table. Details of coverage depth for each *PKP2* exon.**

SD: standard deviation.

| **Exon** | **Patient B.1** | **Patient A.1** | **Patient A.2** | **Patient A.3** | **Patient A.4 (unaffected)** | **Mean coverage of the cohort ± SD** |
| --- | --- | --- | --- | --- | --- | --- |
| **Exon 1** | 5.4 | 10.3 | 9.6 | 10.7 | 10.5 | 9.7±2.1 |
| **Exon 2** | 1.3 | 2.8 | 3.3 | 3.3 | 3.7 | 3.0±1.1 |
| **Exon 3** | 41.0 | 77.8 | 69.3 | 76.0 | 93.9 | 75.4±10.4 |
| **Exon 4** | 23.0 | 22.1 | 15.7 | 25.3 | 45.8 | 38.8±9.5 |
| **Exon 5** | 19.1 | 57.1 | 37.3 | 38.3 | 43.0 | 39.6±7.5 |
| **Exon 6** | 17.6 | 32.7 | 40.9 | 40.4 | 37.9 | 37.3±6.8 |
| **Exon 7** | 21.5 | 54.0 | 48.5 | 50.6 | 47.5 | 47.9±8.5 |
| **Exon 8** | 82.0 | 158.3 | 135.2 | 119.2 | 122.9 | 125.7±16.3 |
| **Exon 9** | 37.0 | 97.3 | 77.0 | 86.7 | 87.7 | 82.1±13.9 |
| **Exon 10** | 29.7 | 68.5 | 57.3 | 63.9 | 76.2 | 61.0±9.5 |
| **Exon 11** | 32.9 | 63.4 | 62.9 | 66.6 | 70.9 | 68.8±10.7 |
| **Exon 12** | 48.9 | 89.8 | 82.4 | 75.0 | 91.7 | 77.1±10.3 |
| **Exon 13** | 62.9 | 141.8 | 117.5 | 149.9 | 158.9 | 136.0±20.1 |
| **Exon 14** | 62.8 | 154.4 | 108.3 | 143.1 | 148.8 | 127.4±18.6 |
