# Supplementary material for: Contribution of exome sequencing for genetic diagnostic in arrhythmogenic right ventricular cardiomyopathy/dysplasia
Source: PLoS One. 2017 Aug 2;12(8):e0181840. doi: 10.1371/journal.pone.0181840 (PMC5540585; doi:10.1371/journal.pone.0181840)
Supplement: S4 Table — Mean ±SD of triplicates. Patient A4 serves as control. (DOCX) [file pone.0181840.s006.docx]

**S4 Table. qPCR values of family A. Mean** ±**SD of triplicates. Patient A4 serves as control.**

|  | **Exon 3** | **Exon 4** | **Exon 5** |
| --- | --- | --- | --- |
| **A1** | **0,93**±0,18 | 0,48±0.04 | 1,07±0,02 |
| **A2** | **0,85**±0,03 | 0,48±0.03 | 0,92±0,03 |
| **A3** | **0,98**±0,1 | 0,48±0.01 | 0,90±0,05 |
| **A4** | **1**±0 | 1±0 | 1±0 |
