# Supplementary material for: Contribution of exome sequencing for genetic diagnostic in arrhythmogenic right ventricular cardiomyopathy/dysplasia
Source: PLoS One. 2017 Aug 2;12(8):e0181840. doi: 10.1371/journal.pone.0181840 (PMC5540585; doi:10.1371/journal.pone.0181840)
Supplement: S5 Table — Mean ±SD of triplicates. Patient A4 serves as control. (DOCX) [file pone.0181840.s007.docx]

**S5 Table. qPCR values of family U. Mean** ±**SD of triplicates. Patient A4 serves as control.**

|  | **Exon 4** | **Exon 13** |
| --- | --- | --- |
| **Control** | 1±0 | 1±0 |
| **U1** | 0.,46±0,05 | 0,62±0,02 |
| **U2** | 0,44±0,07 | 0,59±0,06 |
| **U3** | 0,54±0,06 | 0,52±0,04 |
| **U4** | 1,08±0,14 | 1,00±0,09 |
