# Supplementary material for: Contribution of exome sequencing for genetic diagnostic in arrhythmogenic right ventricular cardiomyopathy/dysplasia
Source: PLoS One. 2017 Aug 2;12(8):e0181840. doi: 10.1371/journal.pone.0181840 (PMC5540585; doi:10.1371/journal.pone.0181840)
Supplement: S7 Table — (DOCX) [file pone.0181840.s009.docx]

**S7 Table. List of CNVs in cardiomyopathy genes detected by the** DNACopy (Bioconductor) package **and frequency of exonic CNV in controls (from data of the database of genomic variants at http://dgv.tcag.ca)**

| **Gene** | **CNV** | **Frequency of exonic CNV in controls (http://dgv.tcag.ca)** | **references** |
| --- | --- | --- | --- |
| *PKP2* | loss (1,02 copy) | 0 |  |
| *ABCC9* | loss (1,35 copy) | 1/17421 ([nsv557744](http://www.ncbi.nlm.nih.gov/dbvar?term=nsv557744))  1/17421 ([nsv557755](http://www.ncbi.nlm.nih.gov/dbvar?term=nsv557755))  1/17421 ([nsv557754](http://www.ncbi.nlm.nih.gov/dbvar?term=nsv557754)) | Cooper et al. 2011 |
| *TNNT2* | gain (3 copy | 1/10 ([nsv947466](http://www.ncbi.nlm.nih.gov/dbvar?term=nsv947466))  1/1557 ([nsv467961](http://www.ncbi.nlm.nih.gov/dbvar?term=nsv467961))  1/17421 ([nsv548997](http://www.ncbi.nlm.nih.gov/dbvar?term=nsv548997))  1/443 ([nsv470776](http://www.ncbi.nlm.nih.gov/dbvar?term=nsv470776)) | Sudmant et al. 2013  Itsara et al. 2009  Cooper et al. 2011  Jacobson et al. 2008 |
| *NEXN* | loss (1.39 copy) | 0 |  |
| *SDHA* | loss (1.4 copy) | Numerous deletions (> 10)  Whole deletion 12/270 ([esv2759315](http://www.ncbi.nlm.nih.gov/dbvar?term=esv2759315)) | Redon et al. 2006 |
| *ACTA1* | gain (3 copies) | 10/10 ([nsv945360](http://www.ncbi.nlm.nih.gov/dbvar?term=nsv945360)) | Sudmant et al. 2013 |
