# Supplementary material for: Contribution of exome sequencing for genetic diagnostic in arrhythmogenic right ventricular cardiomyopathy/dysplasia
Source: PLoS One. 2017 Aug 2;12(8):e0181840. doi: 10.1371/journal.pone.0181840 (PMC5540585; doi:10.1371/journal.pone.0181840)
Supplement: S8 Table — (DOCX) [file pone.0181840.s010.docx]

**S8 Table : Comparison of technique for detection of large PKP2 deletion**

|  | **Advantages** | **Weakness** |
| --- | --- | --- |
| **Exome sequencing** | - Provides information for missense, stop and frameshift mutations at the same time  - Possibility to analyze other gene of interest | - High cost  - Low coverage depth for *PKP2* exon 1 and 2 |
| **Targeted capture** | - Provides information for missense, stop and frameshift mutations at the same time  - Possibility to analyze other gene of interest  - High coverage depth  - Medium cost |  |
| **MLPA** | - High sensitivity (can detect deletions involving only one exon)  - Low cost | - Does not cover all PKP2 exons (exon 1, 2 and 12)  - No information on missense, stop and frameshift mutations |
| **cGH-Array** | - Detailed information on deletion bounds  - Medium cost | - No information on missense, stop and frameshift mutations  - Lacks sensitivity to detect one exon deletion |
| **qPCR** | - Gold standard | - High cost and time consuming  - No information on missense, stop and frameshift mutations  - No information on other genes |
