# Supplementary material for: Contribution of exome sequencing for genetic diagnostic in arrhythmogenic right ventricular cardiomyopathy/dysplasia
Source: PLoS One. 2017 Aug 2;12(8):e0181840. doi: 10.1371/journal.pone.0181840 (PMC5540585; doi:10.1371/journal.pone.0181840)
Supplement: S1 Method — (DOCX) [file pone.0181840.s011.docx]

**S1 Method. Whole exome sequencing and bioinformatic analysis.**

Genomic DNA was captured using the Agilent solution enrichment method with their biotinylated oligonucleotide (Human All Exon, Agilent) probe library, followed by high-throughput sequencing of both ends on 75 b on Illumina HiSEQ 2000.

Sequence capture, enrichment and elution are performed according to the protocol and recommendations of the supplier (SureSelect, Agilent) without modification. Briefly, 3 μg of each genomic DNA is fragmented by sonication and purified to obtain fragments of 150 to 200 bp. The oligonucleotide adapters for the sequencing of the two ends are ligated on the repaired fragments and with an Adenine added at the ends and then purified and enriched by 4 to 6 PCR cycles. 500 ng of these purified libraries are then hybridized to the SureSelect library of capture oligonucleotide probes for 24H. After hybridization, washing and elution, the eluted fraction is amplified by 10 to 12 PCR cycles, purified and quantified by quantitative PCR in order to obtain sufficient template DNA for the rest of the downstream process. Each sample of eluted and enriched DNA is then sequenced on a HiSeq 2000 of Illumina to obtain sequences of 75 b of each end. Images analyses and the determination of bases are carried out by the Illumina RTA version 1.14 pipeline with the default parameters

**Bioinformatic analysis**

The bioinformatic analysis of the sequencing data is based on the pipeline provided by Illumina (CASAVA1.8). CASAVA1.8 is a sequence of scripts including sequence alignment on the complete genome (build37), allele counting and detection of variants (SNPs and Indels). The alignment algorithm used is ELANDv2e (gapped alignment and multi-seed reducing artefactual mismatches). Note that only the positions included in the coordinates of the target regions are retained.

Annotation of genetic variation is performed internally, including gene annotation (RefSeq and Ensembl), referenced polymorphisms (dbsnp132, 1000Genomes) followed by characterization of the mutation (exonic, intronic, silent, missense ....). For each position, exomic frequencies (Homo and HTZ) are also determined, taking into account at least 150 exomes sequenced by IntegraGen. The results are rendered by sample, in the form of tabulated text files. We also provide the result of quality control of targeted sequencing (coverage / depth).

**Variants detection (CASAVA 1.8) :**

- **SNPs:**
  - Process:
  - The software used for the detection of SNPs is the Illumina pipeline. The read pairs are aligned using the ELANDv2 algorithm. The latter allows aligned gears and uses the notion of multi-seeds (a seed = 32nt).
  - The first step is "allele calling": at each position of the genome is determined one or two alleles, associated with a quality score.
  - Only the pairs of reads with an expected insert size (3SD of the median) and a correct orientation (of the Forward-Reverse type) are used during the allele-calling step.
  - Pairs of duplicated reads (strict positions) are excluded from the analysis, indicating an amplification bias during the PCR.
  - The allele-call scores obtained can be transformed into Qphred, multiplying their value by 10. Let an allele score of 3 correspond to a PhredScore of 30.
  - Genotypic status:
    - Homozygous SNPs (the reference allele is not observed) are defined as such when the allele call score is ≥ 6.
    - For heterozygous SNPs, an allele call score ≥ 3 is required for the second allele call score. Moreover, the ratio of their score must be $\leq$ 3.
- **Indels:**
  - Process:
    - The algorithm uses the singleton / shadow reads pairs to detect the indels.
    - Clustering of distant non-aligned shadow-reads from an expected position of their singleton partner
    - Contig assembly
    - Alignment of contigs against the reference genome and counting of the pattern (presence or absence).
- **CNV :**

Analysis is conducted using the DNACopy (Bioconductor) package. The latter makes it possible to detect and localize sudden changes in the number of copies of DNA. The CBS (Circular Binary Segmentation) method of Olshen and Venkatraman (2004) is used to segment the sequencing data of Exome (Circle binary segmentation for the analysis of array-based DNA copy number data.Olshen AB, Venkatraman ES, Lucito R , Wigler M).-The log2 ratio of the reading depth between each sample and a reference pool is calculated (the reference pool depends on the capture kit used). The ratios are standardized by the total number of sequences and adjusted so that the median log2 ratio of the exons of the "normal" chromosomes is zero.
