# Supplementary material for: Contribution of exome sequencing for genetic diagnostic in arrhythmogenic right ventricular cardiomyopathy/dysplasia
Source: PLoS One. 2017 Aug 2;12(8):e0181840. doi: 10.1371/journal.pone.0181840 (PMC5540585; doi:10.1371/journal.pone.0181840)
Supplement: S2 Method — (DOCX) [file pone.0181840.s012.docx]

S2 Method. List of genes known to be associated with inherited cardiomyopathies:

*AARS2, ABCC9, ACTA1, ACTC1, ACTN2, AGK, AKAP9, ANK2, ANKRD1, BAG3, C2ORF64, CACNA1B, CACNA1C, CACNA2D1, CACNB2, CALM1, CALM2, CALR3, CASQ2, CAV3, COX10, COX15, CSRP3, CTNNA3, DES, DSC2, DSG2, DSP, DTNA, EMD, EYA4, FBN1, FHL1, FLNC, GAA GJA5, GLA, GPD1L, HCN4, JPH2, JUP, KCNA5, KCND3, KCNE1, KCNE1L, KCNE2, KCNE3, KCNH2, KCNJ2, KCNJ5, KCNJ8, KCNQ1, KRAS, LAMP2, LMNA, MRPL44, MYBPC3, MYH6, MYH7, MYL2, MYL3, MYLK2, MYOM1, MYPN, NEBL, NEXN, NKX2-5, NPPA, PDLIM3, PKP2, PLN, PRKAG2, PSEN1, PSEN2, PTPN11, RAF1, RANGRF, RBM20, RYR2, SCN1B, SCN2B, SCN3B, SCN4B, SCN5A, SCO2, SDHA, SGCD1, SLC25A4, SNTA1, SOS1, STRN3, SYNPO2, TAZ, TCAP, TGFB3, TMEM43, TMEM70, TMPO, TNNC1, TNNI3, TNNT2, TPM1, TRDN, TTN, TTR, VCL, ZASP_LDB3*
